# Public Perception of Men Who Have Committed Intrafamilial and Extrafamilial Sexual Offences Against Children

**DOI:** 10.1177/10790632211062188

**Published:** 2022-03-08

**Authors:** Megan Hartley, Ross M. Bartels

**Affiliations:** 1 4547 University of Lincoln, Lincoln, UK

**Keywords:** child sexual abuse, intrafamilial, extrafamilial, attitudes, public perception, victim–perpetrator relationship

## Abstract

This study examined whether the attitudinal responses toward child sexual abuse (CSA) differ due to the person’s relationship with the victim (intrafamilial vs. extrafamilial) and/or proximity to the victim (close vs. distant). An online sample of 292 participants completed a measure assessing pre-existing attitudes toward people who commit sexual offenses, before being randomly presented with a vignette describing a CSA case committed by a biological father, biological uncle, babysitter, stranger, or stepfather. Participants then rated the perpetrator’s level of dangerousness and pedophilic interest, their own feelings of disgust, and their punitive judgments. Controlling for pre-existing attitudes, the extrafamilial cases (stranger and babysitter) were perceived to be more dangerous (large effects; *d*s > .50) and more pedophilic than the stepfather (large effects; *d*s > .60). Also, participants reported greater levels of disgust toward the stranger than both the babysitter and uncle (medium effects*; d*s > .30). The findings demonstrate the need to account for the established heterogeneity of men who commit CSA when studying the public’s attitudinal responses. Methodological limitations and suggestions for future research are also discussed.

## Introduction

Much research has been conducted on people’s attitudes toward those who have sexually offended (Harper et al., 2017). This research spans multiple countries, such as Australia (Bartels et al., 2020; Richards, 2011; Shackley et al., 2014), Canada (Sparks, 2021), China (Chui, Cheng, & Ong, 2015), Spain (Marteache, 2012), the UK (Kerr et al., 2018; Sanghara & Wilson, 2006), and the USA (Bowman, 2018; Socia et al., 2019a; Wurtele, 2021), as well as multiple populations, including forensic professionals (Conley et al., 2011; Mellor & Deering, 2010), members of the public (McCoy & Gray, 2007), police officers (Kite & Tyson, 2004), and students (Fuselier et al., 2002). The collective findings from this body of work indicate that, in general, the public hold highly negative attitudes toward individuals who have sexually offended (Harper et al., 2017; Richards & McCartan, 2018). These public attitudes not only affect the well-being of people with sexual convictions due to internalized stigma (Tewksbury, 2012), they also influence legislation and policies, such as the implementation of sex offender registries and residency restrictions (Kernsmith et al., 2009; Mancini et al., 2010), therapeutic efficacy (Beech & Hamilton-Giachritsis, 2005), public vigilantism (Cubellis et al., 2019), jury decisions (Vidmar, 1997), and the successful societal reintegration of people with sexual convictions (Clark, 2007; Willis et al., 2010). Further, it has been argued that problems in each of the above can increase the likelihood of recidivism (Freeman, 2012; Simmons et al., 2021). Thus, it is important that these negative attitudes are understood more deeply. This includes identifying how attitudinal responses are affected by the characteristics of both the offense and the perpetrator (Harper & Bartels, 2018; King & Roberts, 2017). Accordingly, the present study examined people’s attitudinal responses to a man who had committed child sexual abuse (CSA) and whether the responses were affected by the man’s relationship to the victim (i.e., an intrafamilial vs. extrafamilial offense) and their proximity to the victim (i.e., close vs. distant).

### Attitudes toward people who have committed CSA

Attitudes are defined as positive or negative evaluations of a person, group, object, or entity (Ajzen & Fishbein, 1977). However, as Breckler (1984) notes, “to say a researcher is measuring ‘attitude’ is ambiguous” (*p*. 1203). This is because attitudes are composed of three distinguishable components that can manifest in response to an attitude object (Breckler, 1984; Rosenberg et al., 1960). This includes a cognitive component (e.g., beliefs about an attitude object), an affective component (e.g., emotional reactions toward an attitude object), and a behavioral component (i.e., overt actions related to an attitude object, such as voting behavior, as well as self-reported behavioral preferences or intentions). Each component reflects the evaluative judgment of an attitude object and has been examined with regards to people who have sexually offended against children.

Regarding the cognitive component, the public tend to believe that people who commit CSA are dangerous because of a high reoffending rate (Fortney et al., 2007) and a resistance to treatment (Richards & McCartan, 2018). However, the sexual recidivism rate is lower than expected (Levenson et al., 2010; Sample & Bray, 2006), with 5.6% [95% CI 4.8–6.5] receiving a 5-year predicted recidivism median score of 2 (average risk) on the Static-99R (Hanson et al., 2017). The public also tend to believe that CSA is synonymous with “pedophilia” (Richards, 2018). However, these are distinct concepts. Fewer than half of the people who commit CSA are pedophilic (Schmidt et al., 2013), and those who are pedophilic are not destined to offend (Cantor & McPhail, 2016). Nevertheless, this misunderstanding has caused “pedophile” to become a highly stigmatized term (Imhoff, 2015; Jahnke et al., 2015a, 2015b). A third public belief is that CSA tends to be perpetrated by a stranger (Levenson et al., 2007; Pickett et al., 2013; Socia & Harris, 2016). This belief (often referred to as “stranger danger”) depicts CSA as being committed by an unknown man who preys on random children (Chenier, 2012; King & Roberts, 2017). However, CSA is typically perpetrated by someone known to the victim, as indicated by research and reports from the UK (Office for National Statistics, 2020), USA (National Institute of Justice, 2003), Canada (Cotter & Beaupré, 2014), Spain (Ferragut et al., 2021), Australia (Australian Bureau of Statistics. 2017), and China (Girl Protection, 2017). Further, trained forensic professionals tend to rate parents and stepparents as being a more likely perpetrator of CSA (Fuselier et al., 2002).

Regarding the affective component, women report greater negative affect toward people who have sexually offended compared to men (Willis et al., 2013). Bastian et al. (2013) found that reading about CSA elicited greater feelings of anger, contempt, and disgust relative to non-sexual offenses. The behavioral component is thought to be expressed through ratings of social distance, which tend to be greater in women compared to men (Deluca et al., 2018; Willis et al., 2013). Also, since punitiveness is regarded as “the level of support individuals demonstrates toward concepts and ideas that promote retribution, incapacitation, and deterrence practices” (Gideon & Sherman-Oren, 2014, *p*. 152), self-reported support for harsher sentences could be construed as an expression of the behavioral component. This includes showing support for punitive practices and policies, such as sex offender registries (Bartels et al., 2020; Comartin et al., 2009; Socia et al., 2019b). Research indicates that people more strongly support harsher sentences for sexual offenses relative to non-sexual offenses (Rogers & Ferguson, 2011) and for those convicted of CSA relative to those with adult victims (Socia et al., 2019b).

Research also indicates that the cognitive and affective components influence punitive judgments. For example, stronger endorsement of stereotypical beliefs about CSA (cognitive component) is associated with greater support for harsher sentencing (Pickett et al., 2013; Sparks, 2021; Wurtele, 2018) and for residence restrictions (Budd & Mancini, 2016). Further, the endorsement of harsher sentencing is greater when the attitude object is more representative of the “sex offender stereotype” (Harper & Bartels, 2018; King & Roberts, 2017; Levenson et al., 2007; Mancini et al., 2010). In terms of the affective component, Bastian et al. (2013) found that greater feelings of disgust toward CSA were associated with greater dehumanization and stronger support for harsher sentences.

These findings pertaining to the cognitive component highlight the disconnect between what the public believes about CSA and what is known from empirical research. These misconceptions, alongside negative affective reactions, influence people’s behavioral responses and punitive judgments. However, people who commit CSA are not a homogenous group. Instead, they differ in terms of their motivation to offend, their historical and personal characteristics, and the specific offending behaviors they engage in. One question that has not received much attention is whether the public’s responses to CSA is affected by the subtype of CSA presented. One common approach to categorizing CSA subtypes is based on the perpetrator–victim relationship (Davies & Rogers, 2009; McCoy and Gray, 2007), which the current study focuses on.

### Intrafamilial and Extrafamilial CSA

Intrafamilial CSA refers to the sexual abuse of a child within the family, whether the relationship is biological or sociolegal (e.g., a stepparent). Extrafamilial CSA, on the other hand, indiscriminately refers to offenses committed against a child outside of the family unit (Fischer & McDonald, 1998). Marshall et al. (2015) added that context and motives are also important to consider when categorizing people who commit extrafamilial CSA. They proposed the term “affiliative” to describe those who abuse children in their care or who they know. This includes professionals, such as teachers and babysitters. According to Marshall et al. (2015), those classed as affiliative are categorically different in the tactics that they utilize (e.g., grooming). By contrast, “non-affiliative” refers to offenses committed by strangers. Those classed as non-affiliative are more likely to be dangerous (in terms of use of violence) as they lack an emotional connection to the child (Marshall et al., 2015). Furthermore, perpetrators with extrafamilial male victims (35% after 15 years) and female victims (16% after 15 years) are more likely to reoffend than incest offenders (13% after 15 years) (Harris & Hanson, 2004). Indeed, on the Static-99R (Hanson & Thornton, 2000), a “stranger victim” (defined as knowing the victim for less than 24 hours prior to the offense) is included as a factor (item) for assessing reoffending risk. Based on these propositions, the current study incorporated this affiliative dimension into the design to examine whether the public perceive non-affiliative offenders as more dangerous.

Much research has examined the difference between those who commit intrafamilial and extrafamilial CSA (Fischer & McDonald, 1998; Pullman et al., 2017; Seto et al., 2015). Findings indicate that those who have committed extrafamilial offenses tend to be more sexually aroused by children (Rice & Harris, 2002; Seto et al., 2015) and more emotionally congruent with children (McPhail et al., 2013) compared to their intrafamilial counterparts. According to Seto et al.’s (2015) meta-analysis, individuals with extrafamilial offenses also have a higher risk of reoffending, indicated by higher scores on the Static-99R (Hanson & Thornton, 2000). In terms of distinguishing biological and sociolegal intrafamilial offenders, a recent meta-analysis found no differences in paraphilic interest levels (Pullman et al., 2017). However, those in a sociolegal position typically presented with greater self-regulation and impulsivity problems than a biological position (e.g., a stepparent vs. biological parent).

### Public Perception of CSA subtypes

Very little research has examined whether the public’s perception of extrafamilial and intrafamilial CSA matches what the literature tells us. The literature demonstrates that men who commit extrafamilial CSA are typically more dangerous and pedophilic than those who commit intrafamilial offenses. However, the public may instead view intrafamilial offenders with more disgust and as more pedophilic than extrafamilial offenders as they have engaged in incestuous behavior, which people are “supposed to” innately avoid as suggested by the Westermarck Effect hypothesis (Westermarck, 1891)

The Westermarck Effect hypothesis states that genetically close individuals, who live in close proximity during childhood, develop sexual aversion to one another in later life, leading to incest avoidance (Walter & Buyske, 2003). Based on Pullman et al.’s (2019) recent study examining the Westermarck hypothesis, sexual aversion may be influenced by feelings of disgust, which they argued may act as a proximate mechanism for preventing parent–child incest. Other research also indicates that feelings of disgust extend beyond one’s own involvement in the incestuous behavior and differs depending on the familial relationship (biological vs. sociolegal incest). For example, Antfolk, Karlsson, et al. (2012) found that biological incest was viewed with more disgust than unrelated incest. Furthermore, disgust ratings for third-party incest are stronger if the raters themselves are related to one of the individuals (Antfolk et al., 2012, 2018).

A few other studies have examined public perceptions of CSA subtypes. For example, Reynolds and Birkimer (2002) found that CSA committed by a stepfather was rated as more harmful than CSA committed by a neighbor. Using a similar design to the present study, Davies and Rogers (2009) compared students’ perceptions of CSA committed by a father and a stepfather. They also manipulated the emotional closeness of the perpetrator–victim relationship, arguing that emotionally close perpetrators may be viewed more harshly due to the greater betrayal of trust. They found a small effect showing that CSA was perceived as more severe when perpetrated by the victim’s father compared to stepfather (η_p_^2^ = 0.03). They also found a medium effect on interaction between the victim–perpetrator relationship and emotional closeness, whereby the CSA was perceived as more severe if perpetrated by an emotionally close biological father (η_p_^2^ = 0.06) relative to an emotionally distant one. Although Davies and Rogers (2009) did not include an extrafamilial condition, their findings support the need to compare perceptions of a biological father and a stepfather. They also provide a rationale for including “emotional closeness” as a factor in research on this topic.

### Present study

The present study aimed to determine whether people’s attitudinal responses toward a man who has committed CSA are influenced by: (1) the perpetrator–victim relationship (i.e., intrafamilial vs. extrafamilial); and (2) the degree of closeness between the perpetrator and victim. Based on existing literature, it can be argued that intrafamilial CSA, relative to extrafamilial CSA, will elicit greater negative affect-based responses (i.e., greater disgust), more stereotypical cognitive-based perceptions (i.e., perceiving the perpetrator to be more dangerousness and more pedophilic), and more negative behavioral-based responses (i.e., stronger support for punishment). On the other hand, it is possible that the extrafamilial CSA cases will be viewed as more dangerous given the belief that CSA is largely committed by pedophilic strangers. Further, it can be argued that negative responses will also be influenced by whether the CSA was committed by someone who is emotionally closer to the child rather than someone who has a more distant relationship. For example, a babysitter and stranger would likely be perceived differently despite both being extrafamilial. This reflects Marshall et al.’s (2015) proposed affiliative dimension. Also, as Seto et al. (2015) suggest, and Davies and Rogers (2009) demonstrated, we distinguished between stepfathers and biological fathers to determine whether perceptions differ depending on the whether the incestuous offense is biological or sociolegal in nature. This study also conceptually extends upon the research by Antfolk et al. (2012, 2018) as they focused on parental relationships, while we have included a distant familial relationship (i.e., an uncle) and a close non-familial relationship (i.e., a babysitter). Also, unlike Antfolk et al. (2012a), we focused male perpetrators (e.g., father–child incest), rather than parent–child incest more broadly.

Based on the above, the following hypotheses were tested:

1. Disgust ratings will be higher for the intrafamilial cases (Father, Stepfather, and Uncle) compared to the extrafamilial cases (Babysitter and Stranger).
2. Extrafamilial cases, particularly the Stranger case, will be judged harsher than intrafamilial cases (i.e., greater punitive and dangerousness ratings).
3. There will be a significant difference between intrafamilial and extrafamilial conditions on the perceived level of sexual interest in children. No specific direction was hypothesized as the public tend to believe that CSA in general constitutes a pedophilic interest (McCartan, 2010).

## Method

### Participants

A Qualtrics survey link was sent out on social media sites, the university’s participant recruitment system, and shared through word-of-mouth. In total, 363 people started the study. After removing those who did not fully complete it, a final opportunity sample of 292 participants was left (the completion rate was 80.4%). The mean age of participants was 30 years old (*SD* = 12.63), with an age range of 18–73 years old. Fifty-six participants identified as male, 234 identified as female, and two identified as non-binary. Most of the sample identified as White/Caucasian (88%). See Table 1 for further demographic details. The participants were randomly assigned to one of the five conditions (see *Design*): “Biological Father” (*n* = 57), “Stepfather” (*n* = 58), “Uncle” (*n* = 60), “Babysitter” (*n* = 55), and “Stranger” (*n* = 62).

**Table 1.** Demographic Information of the Sample Separated by the Five Different Conditions.

	Father (*n =* 57)		Stepfather (*n* = 58)		Uncle (*n* = 60)		Babysitter (*n* = 55)		Stranger (*n* = 62)	
	*n*	*%*	*N*	*%*	*n*	*%*	*n*	*%*	*n*	*%*
*Gender*										
Male	12.0	21.1	10.0	17.2	10.0	16.7	13.0	23.6	11.0	17.7
Female	43.0	75.4	48.0	82.8	50.0	83.3	42.0	76.4	51.0	82.3
Non-binary	2.0	3.5	-	-	-	-	-	-	-	-
***Relationship Status***										
Single	24.0	42.1	20.0	34.5	20.0	33.3	20.0	36.4	28.0	45.2
In a relationship	33.0	57.9	38.0	65.5	40.0	66.7	34.0	61.8	34.0	54.8
***Job Status***										
Student	41.0	71.9	36.0	62.1	39.0	65	37.0	67.3	47.0	75.8
Full-time employed	13.0	22.8	19.0	32.8	19.0	31.7	11.0	20.0	15.0	24.2
Retired	1.0	1.8	2.0	3.4	1.0	1.7	5.0	9.1	-	-
Unemployed	2.0	3.5	1.0	1.7	-	-	-	-	-	-
***Parental Status***										
Parent	10.0	17.5	10.0	17.2	13.0	21.7	11.0	20.0	12.0	19.4
Non-parent	47.0	82.5	47.0	81	47.0	78.3	44.0	80.0	49.0	79.0
***Related to a Child?***										
Yes	38.0	66.7	47.0	81.0	43.0	71.7	44.0	80.0	50.0	80.6
No	15.0	26.3	7.0	12.1	12.0	20.0	7.0	12.7	10.0	16.1
***Age (in years)***										
Mean	25.3		29.3		27.9		30.0		27.5	
Standard Deviation	9.9		14.5		11.9		14.13		12.1	
Range	18–62		18–73		18–59		18–67		18–60	

## Materials

*Attitudes Toward Sex Offenders Scale – 21 (ATS-21;* Hogue & Harper, 2019*)*: Pre-existing attitudes toward people who have sexually offended were measured using the ATS-21. This is a shortened version of the original 36-item ATS (Hogue, 1993). Participants rate how much they agree with the 21 attitudinal statements using a 5-point Likert scale, ranging from 0 (*Strongly* disagree) to 4 (*Strongly* agree). Scores range from 0–84, with higher scores indicating more positive attitudes. The ATS-21 consists of three subscales, which, in the present study, showed very good Cronbach’s alphas: “Trust” (7 items, α = .82), “Intent” (7 items, α = .85), and “Social Distance” (7 items, α = .82). The ATS-21 total had an excellent internal consistency (α = .93).

*Vignettes:* Five vignettes, each depicting a case of child sexual abuse, were developed for this study. These vignettes were near-identical, in that, all fives offenses were perpetrated by a 30 year-old man named Charles, and all took place in the child’s bedroom. Slight differences in certain contextual elements were necessary to explain the perpetrator’s presence in the home. The exact age and gender of the child was left ambiguous in the vignette (see Appendix 1). The two factors manipulated across the vignettes were: (1) relationship type (related or non-related) and (2) relationship proximity (close or distant). This allowed us to examine participants’ perceptions of an offense committed by a distant intrafamilial man (uncle); a close intrafamilial man (biological father); a distant extrafamilial man (stranger); and an extrafamilial but close man (babysitter). This final category (babysitter) reflects Marshall et al.’s (2015) concept of the affiliative perpetrator. The final vignette involving a stepfather could not be suitably broken down in the context of the manipulated two factors.

*Attitudinal responses:* Following the vignette, the participants completed a series of questions rated on a 7-point scale. One cognitive-based question assessed dangerousness: “*To what extent do you think Charles poses a danger to other children*?” (1 = “No danger” to 7 = “Extremely dangerous”). A second cognitive-based question asked about Charles’ level of pedophilic interest: “*To what extent do you think Charles has a sexual interest in children*?” (1 = “Not at all” to 7 = “A complete preference for children”). Note, for this question, the term “pedophilia” was not used as it has been shown to heighten negative views (Imhoff, 2015). The third was an affect-related question assessing their feeling of disgust: “*As a result of reading this interaction, to what extent did you feel physically disgusted*?” (1 = “Not at all” to 7 *=* “Extremely”).

*Punitive Judgments:* To gauge participants’ punitive judgments about man in the vignette (Charles), we used a 7-item version of Imhoff’s (2015) punitive attitudes scale. However, like Jahnke (2018), we adapted the seven items so that they referred specifically to Charles, rather than men in general who have sexually abused a child (e.g., “*People like Charles should be castrated*”). These seven items were rated on a 7-point Likert scale ranging from 1 (“Do not agree at all”) to 7 (“Completely Agree”). A mean score was calculated for each participant, with higher scores indicating stronger punitive judgments. In the present study, the Cronbach’s alpha for this measure was very good (α = .82).

### Design

We developed an online vignette study using a between-subjects experimental design that involved the following two factors: *Relationship Type* (biologically related vs. unrelated) and *Relationship Proximity* (close vs. distant; see Appendix 1 for vignettes). Participants were randomly allocated to one of five conditions. Four of these conditions were based on the perpetrator–victim relationship present within the vignette: namely, Biological Father (Close, Related), Uncle (Distant, Related), Babysitter (Close, Unrelated), Stranger (Distant, Unrelated). The fifth condition involved a vignette depicting CSA committed by a Stepfather, which did not fit the two factors. The dependent variables were judgments of dangerousness, level of pedophilic interest, level of felt disgust, and punitive judgments. An assessment of pre-existing attitudes toward people who have sexually offended was also included as a covariate.

### Procedure

After clicking on the Qualtrics link, participants were provided with an Information Sheet explaining the purpose of the study, what they had to do, and their various rights. Full consent was required to continue. Demographic questions were then completed, followed by the ATS-21. Participants were then randomly allocated into one of the five vignette conditions. After reading their assigned vignette, participants provided their attitudinal responses about Charles. Following this, participants were asked whether they were a parent or held any type of close relationship with a child. A full debrief was then shown providing information about the purpose of the study and the different conditions involved. Participants were signposted to helplines should they have been adversely affected by anything during the study. On average, the study took approximately 15 minutes to complete. Students recruited via the university’s participation system were awarded one credit for partaking in the study.

### Data Analysis

Two separate MANCOVAs were run to test the three hypotheses. Since the stepfather condition could not be suitably broken down into the two separate factors (i.e., Relationship Type and Relationship Proximity), the first test was a one-way independent MANCOVA, which tested the difference in attitudinal responses between the five conditions. “Condition” was entered as the between-subjects factor, with “punitive judgments,” “perceived pedophilic interest,” “perceived dangerousness,” and “disgust” entered as the dependent variables. The ATS-21 was entered as the covariate (see below). Although the Levene’s tests were non-significant for “punitive judgments” (*F* (4,283) = 1.80, *p* = .129, “perceived pedophilic interest” (*F* (4,283) = 0.02, *p* = .999), “perceived dangerousness” (*F* (4,283) = 2.19, *p* = .070), and “disgust” (*F* (4,283) = 1.23, *p* = .300), the sensitive Box’s M test was significant (Box M = 58.55, *F* = 1.42, *p* = .042). Thus, Pillai’s trace was used instead of Wilk’s Lambda, as Pillai’s trace is more robust to violations of model assumptions (Olson, 1976).

The second test was a 2 (*Relationship Type*: related vs. unrelated) x 2 (*Relationship Proximity*: close vs. distant) independent MANCOVA, using the same dependent variables and ATS-21 scores as a covariate. This enabled us to examine the potential interaction between Relationship Type and Relationship Proximity. Data from the Stepfather condition was not included in this two-way MANCOVA. Again, the Levene’s Tests were non-significant for all of the dependent variables: “punitive judgments,” *F* (3,227) = 2.16, *p* = .09, “perceived pedophilic interest,” *F* (3,227) = 0.01, *p* = .99, “perceived dangerousness,” *F* (3,227) = 1.84, *p* = .14, and “disgust,” *F* (3,227) = 1.32, *p* = .27, but the Box’s M was significant (Box’s M = 51.94, *F* = 1.68, *p* = .012). Thus, Pillai’s trace was used again. The follow-up pairwise comparisons are reported alongside the relevant hypothesis in the Results section. Effect sizes were interpreted according to Cohen’s (1988) guidelines, whereby a small η_p_^2^ = .01, a medium η_p_^2^ = .06, and a large η_p_^2^ = .14, and a small Cohen’s *d* = 0.2, a medium = 0.5, and a large = 0.8.

## Results

### Demographic differences

Due to participants being randomly assigned to the five experimental conditions, we tested for any demographic differences. Note, for the chi-square analyses, Cramer’s V (ϕc) indicates the effect size, whereby a value of 0.1 is weak, 0.3 is medium, and larger than 0.5 is strong (Cohen, 1988). No differences were found with respect to participant gender (*χ*^2^ (8) = 9.71, *p* = .286, ϕc = .29), age (*F* (4,290) = 1.17, *p* = .324, η_p_^2^ = .016, small effect), relationship status (*χ*^2^ (4) = 2.60, *p* = .628, ϕc = .63), job status (*χ*^2^ (16) = 22.37, *p* = .131, ϕc = .13), parental status (*χ*^2^ (4) = 0.46, *p* = .977, ϕc = .98), other familial links to children (*χ*^2^ (8) = 7.22, *p* = .513, ϕc = .51), and the ATS-21 total (*F* (4,291) = 0.49, *p* = .742, η_p_^2^ = .007, small effect). See Table 2 for descriptive statistics across conditions.

**Table 2.** Means and Standard Deviations for the Covariate (ATS-21) and Outcome Variables Across the Five Conditions.

	Father (Related, Close)		Uncle (Related, Not close)		Stepfather		Babysitter (Unrelated, Close)		Stranger (Unrelated, Not close)	
Variable	*M*	*SD*	*M*	*SD*	*M*	*SD*	*M*	*SD*	*M*	*SD*
ATS-21 total	34.74	13.06	33.48	14.16	36.62	14.16	33.96	13.74	35.47	12.64
Punitive judgement	4.34	1.17	4.43	1.27	4.17	1.22	4.42	1.33	4.53	1.20
Sexual interest in children	5.43	1.06	5.78	1.09	5.12	1.07	5.93	0.94	5.85	0.93
Level of disgust	6.14	1.32	6.07	1.22	5.88	1.16	5.91	1.30	6.41	0.94
Level of dangerousness	5.96	1.18	6.17	1.19	5.77	1.23	6.40	0.99	6.44	0.87

*Note.* For the ATS-21, higher scores indicate more positive attitudes toward people who have sexually offended.

### Correlation between ATS-21 and outcome questions

Pearson’s correlation analyses revealed that the ATS-21 was significantly associated with punitive judgments (*r* = −.62, *p* <.001), perceived pedophilic interest (*r* = −.23, *p* <.001), perceived dangerousness (*r* = −.41, *p* <.001), and disgust ratings (*r* = −.31, *p* <.001). Given its correlation with each attitudinal response, the ATS-21 total was entered as a covariate into the subsequent analyses so that any differences between conditions were not due to pre-existing attitudes (Harper et al., 2017, 2018).

### Hypothesis Testing

The one-way MANCOVA revealed a statistically significant difference (but small effect) between the five conditions on the dependent variables, after controlling for ATS-21 scores, *F* (16,1128) = 2.68, *p* < .001, Pillai’s trace = .146, η_p_^2^ = .037. Regarding the two-way MANCOVA, the results revealed no significant main effect of “Relationship Proximity,” *F* (4,223) = 0.57, *p* = .688, Pillai’s trace = .01, η_p_^2^ = .01. However, there was a significant moderate main effect of “Relationship Type,” *F* (4,223) = 3.15, *p* = .015, Pillai’s trace = .054, η_p_^2^ = .054. Moreover, there was a moderate significant interaction effect, *F* (4,223) = 3.38, *p* = .010, Pillai’s trace = .057, η_p_^2^ = .057. Post-hoc tests are reported alongside the corresponding hypotheses below.

Hypothesis 1 Disgust will be stronger in the intrafamilial cases (Father, Stepfather, and Uncle) than the extrafamilial cases (Babysitter and Stranger).

**Figure 1.**
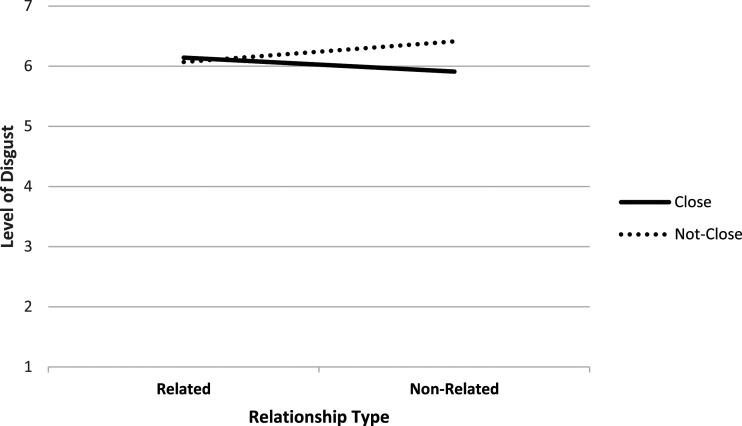
Interaction between Relationship Proximity and Relationship Type on self-reported level of disgust.

Post-hoc results from the one-way MANCOVA showed a non-significant effect of Condition on “disgust,” *F* (4,282) = 2.19, *p* = .071, η_p_^2^ = .03. However, post-hoc analyses of the two-way MANCOVA revealed no main effects of Relationship Proximity, *F* (1,231) = 2.02, *p* = .157, η_p_^2^ = .009, or Relationship Type, *F* (1,231) = 0.33, *p* = .565, η_p_^2^ = .001. However, there was a small significant interaction effect, *F* (1,231) = 6.21, *p* = .031, η_p_^2^ =.021 (see Figure 1). Simple main effects analysis (with Bonferroni correction) revealed that disgust ratings were significantly greater in the Stranger condition (unrelated, not close) than in the Babysitter condition (unrelated, close) (*p* = .012, *d* = 0.44, small effect). Moreover, disgust levels in the Stranger condition (not close, unrelated) were higher than in the Uncle condition (not close, related) (*p* = .048, *d* = 0.36, small effect). Therefore, Hypothesis 1 is rejected as disgust was greater for the Stranger (extrafamilial) case than the Uncle (intrafamilial) case.

Hypothesis 2 Extrafamilial cases, particularly the Stranger case, will be judged harsher than intrafamilial cases (i.e., higher punitive judgment and dangerousness scores).

For the one-way MANCOVA, post-hoc analyses (with Bonferroni correction) revealed no main effect of Condition on punitive judgments, *F* (4,282) = 0.69, *p* = .603, η_p_^2^ = .01). However, a significant moderate main effect of Condition on “dangerousness” was found, *F* (4,282) = 4.19, *p* = .003, η_p_^2^ = .056. Specifically, the Stepfather was perceived to be less dangerous than both the Babysitter (*p* = .048, *d* = 0.56, medium effect) and Stranger (*p* = .007, *d* = 0.63, medium effect). The difference between the Father and Stranger conditions was approaching significance (*p* = .056, *d* = 0.46), with the Father perceived as less dangerous than the Stranger.

Post-hoc analyses from the two-way MANCOVA for “punitive judgments” showed no main effect of Relationship Proximity, *F* (1,231) = 0.81, *p* = .369, η_p_^2^ = .004, or Relationship Type, *F* (1,231) = 0.97, *p* = .326, η_p_^2^ = .004, and no interaction effect, *F* (1,231) = 0.23, *p* = .635, η_p_^2^ = .001. Regarding “dangerousness,” the two-way MANCOVA revealed no main effect of Relationship Proximity, *F* (1,231) = 1.07, *p* = .302, η_p_^2^ = .005, and no interaction effect, *F* (1,231) = 1.08, *p* = .742, η_p_^2^ = .00. However, there was a significant, small-medium main effect of Relationship Type, *F* (1,231) = 8.67, *p* = .004, η_p_^2^ = .037. That is, extrafamilial men were rated as being more dangerous to other children than those in the intrafamilial conditions (*p* = .004, *d* = 0.33, small effect). Therefore, Hypothesis 2 is partially accepted as unrelated (extrafamilial) cases were deemed more dangerous than related (intrafamilial) cases.

Hypothesis 3 There will be a significant difference between intrafamilial and extrafamilial conditions on the perceived level of sexual interest in children.

Post-hoc analyses (with Bonferroni correction) from the one-way MANCOVA found a significant and strong main effect of Condition on “perceived pedophilic interest” ratings, *F* (4,282) = 5.98, *p* < .001, η_p_^2^ =.078. Participants in the Uncle (*p* = .019, *d* = 0.61, medium effect), Babysitter (*p* < .001, *d* = 0.80, large effect), and Stranger (*p* < .001, *d* = 0.73, medium-large effect) conditions reported higher ratings relative to those in the Stepfather condition. For the two-way MANCOVA, post-hoc analyses revealed a significant main effect of Relationship Type on “perceived pedophilic interest,” *F* (1,231) = 5.75, *p* = .017, η_p_^2^ = .025. That is, men in the unrelated cases were rated as having a greater sexual interest in children than men in the related cases (*p* = .017, *d* = 0.28, small effect). There was no significant main effect of Relationship Proximity (*F* (1,231) = 1.04, *p* = .308, η_p_^2^ = .005) and no interaction effect, *F* (1,231) = 1.83, *p* = .178, η_p_^2^ = .008.

## Discussion

The present study examined whether the public’s attitudinal responses toward a man who had committed child sexual abuse (CSA) were affected by the perpetrator–victim relationship. Specifically, controlling for pre-existing attitudes toward people who have sexually offended, we investigated whether Relationship Type (extrafamilial vs. intrafamilial) and Relationship Proximity (close vs. distant) influenced participants’ ratings of dangerousness, perceived pedophilic interest, disgust, and punitive judgments. These attitudinal responses reflect the three components underlying attitudes (Breckler, 1984). That is, perceived dangerousness and pedophilic interest reflected the cognitive component, disgust reflected the affective component, and punitive judgments (we argue) reflected the behavioral component. The findings supported two out of our three hypotheses.

### Feelings of disgust

Drawing upon research on incest aversion and incest taboo (Antfolk et al., 2012a), we first hypothesized that intrafamilial cases of CSA (i.e., committed by a father, uncle, or stepfather) would elicit more disgust than extrafamilial cases (i.e., those committed by a stranger or babysitter). Contrary to expectation, disgust ratings were higher for CSA committed by an extrafamilial stranger (unrelated, distant) compared to the corresponding intrafamilial case committed by an uncle (related, distant). This medium difference (*d* = 0.36) suggests that, although disgust responses may be influenced by incest aversion when responding to an intrafamilial case, other factors play a more potent role when responding to extrafamilial CSA. It could be argued that greater disgust responses are elicited by CSA cases that match stereotypical representations (Rydberg et al., 2018; Socia et al., 2019b). That is, the public tends to believe that perpetrators of CSA are strangers (Pickett et al., 2013). Indeed, disgust ratings were greater in the stranger condition compared to the babysitter condition to a moderate-to-high degree (*d* = 0.44). Therefore, when activated, the stereotypes people hold about CSA may lead to stronger feelings of disgust than incest aversion.

However, it should be noted that out findings also conflicted with Antfolk et al. (2012a). In their study, they found that a biologically related parent (intrafamilial) elicited more disgust than an unrelated parent (extrafamilial), while we found no differences in disgust between the equivalent conditions (Father vs. Stepfather). This discrepancy could be accounted for by the fact that our findings pertain to male perpetrators, whilst Antfolk et al. (2012a) analyzed parent–child incest more broadly (male and female perpetrators combined).

Furthermore, Antfolk et al. (2012a) used a student sample, whereas our sample was a mix of students and non-students. This may have accounted for the discrepant findings. Another possible reason for the cross-study differences could be attributed to the fact that we compared vignettes using a between-subjects design, whilst Antfolk et al. (2012a) did so using a within-subjects design. This may indicate a contrast effect was at play in their study. That is, it is possible that people experience greater disgust toward biological incest when directly compared to non-biological incest, as opposed to when disgust is compared across participants. Future research is, therefore, needed to examine these possibilities in order to help resolve the inconsistency between our two studies.

### Perceived dangerousness

In line with Hypothesis 2, men who were unrelated to the victim were perceived to be more dangerous than those who were related. According to the one-way MANCOVA, the stranger (*d* = 0.63), followed by the babysitter (*d* = 0.56), were rated as posing more of a danger to other children than the stepfather (who was rated as least dangerous). As shown, the magnitude of these differences was strong. These findings, alongside those regarding disgust, could be explained by a common belief held by the public (Chenier, 2012), wherein children are seen as being most at risk of being sexually abused by an extrafamilial person (particularly a stranger). However, sexual abuse victims are statistically more likely to know the person who abused them (NIJ, 2003; ONS, 2020). Thus, this belief may have led to the greater dangerousness and disgust responses. However, it should be noted that, if we frame “dangerousness” as being “risk of recidivism,” then the current results are in line with the literature. That is, men who commit extrafamilial CSA are more likely to sexually reoffend compared to those who commit intrafamilial offenses (Hood et al., 2002). Future research should examine the judgments of dangerousness in a more nuanced manner to ascertain whether it reflects a *Stranger Danger* belief or a view regarding reoffending risk.

### Perceived pedophilic interest

The third hypothesis related to Charles’ perceived level of pedophilic interest. Our two-tailed prediction stated that there would be a significant difference between the extrafamilial and intrafamilial cases. The mean rating across conditions was above the mid-point, suggesting that most participants judged Charles to have a relatively strong sexual interest in children. This aligns with prior research showing that the public often conflate CSA and pedophilia (McCartan, 2010; Richards, 2018). There was, however, some variation. For example, the stepfather received the lowest rating, while the babysitter received the highest rating. Statistical analyses revealed that the stranger (*d* = 0.73), babysitter (*d* = 0.80), and uncle (*d* = 0.61) were all judged as having a statistically significant stronger pedophilic interest than the stepfather. As indicated, the magnitudes of these differences were strong, particularly for the stranger and babysitter. The results of the two-way MANCOVA (which did not include the stepfather condition data) showed that extrafamilial men were perceived to harbor a statistically significant stronger pedophilic interest than intrafamilial perpetrators. However, the magnitude of this particular effect was much smaller (*d* = 0.28). Collectively, these findings support Hypothesis 3 and suggest that the public’s view of pedophilia and CSA aligns with empirical research, as extrafamilial offenses have been found to be associated with pedophilic sexual interest (Seto et al., 2015; Seto & Lalumière, 2001). However, it is unlikely that the public are aware of this literature. Thus, future research should look to determine why people who commit extrafamilial CSA are viewed as being more pedophilic.

### Punitive judgments

Unexpectedly, no differences were found for punitive judgments. The influence of implicit theories may account for this finding. As conceptualized by Dweck et al. (1995), implicit theories are beliefs that guide how people interpret and respond to the actions of others. These can be incremental beliefs, whereby behavior is seen as changeable (e.g., through effective treatment), or entity beliefs, where behavior is perceived to be resistant to change (Dweck et al., 1995). Implicit theories have been shown to be related to how people respond to people who have sexually offended (Harper & Bartels, 2017). For example, those with entity-based implicit theories make more punitive judgments about people who have sexually offended compared to those with more incremental implicit theories (Harper & Bartels, 2018). Thus, many of the participants in the current study may have held entity-based implicit theories about people who commit CSA, leading them to rate Charles more punitively regardless of his relationship to the child. This would account for the lack of significant differences between the conditions. Future researchers should, therefore, take account for these beliefs, either by examining their moderating effect or entering them as covariates in the analysis.

### Limitations

The present study has offered some useful insights into the public’s view of CSA and the men who commit such offenses. However, several limitations are worth highlighting. For example, the sample was comprised of more self-identified female participants than male participants (56 identified as male, 234 identified as female, and 2 as non-binary). This may have biased the findings, as female participants have been shown to judge sexual assault as more severe, the perpetrator more culpable (Rogers & Davies, 2007) and rate third-party incest as more disgust eliciting than male counterparts (Antfolk et al., 2012a). A second issue was the inability to suitably categorize the stepfather condition into the two separate factors of the two-way MANCOVA (i.e., Relationship type and Relationship proximity). That is, the stepfather could have been coded as being unrelated but close to the child. However, this was how the babysitter was coded. Alternatively, the stepfather could have been coded as being related (in a sociolegal manner) and close to the child. However, this is how we coded the biological father. As a result, the stepfather data was omitted from the two-way MANCOVA. Nevertheless, the one-way MANCOVA allowed us to directly compare the father and stepfather condition to ascertain whether perceptions of CSA, particularly disgust, were driven by an aversion to incest. Contrary to Davies et al.’s (2013) results, we found that the father-perpetrated CSA was *not* viewed as more severe than stepfather-perpetrated CSA. Future studies should add a “distant stepfather” condition so that a direct comparison with the close stepfather can be made.

A third point is that the age and gender of the child was not overtly stated in the vignettes. Thus, future research should aim to compare pre- and post-pubescent victims and female/male victims (Davies et al., 2010; Socia et al., 2019). Additionally, any replications of the present study should include a way to account for careless and random responding (Goldammer et al., 2020). Also, the use of single-items to assess dangerousness, the level of pedophilic interest, and disgust has limitations. As mentioned earlier, the term “dangerous” could be interpreted differently across participants (e.g., use of violence, likelihood to offend, likelihood of reoffend). Thus, future studies should aim to use multi-item measures, as they cancel out random measurement error and can reduce idiosyncratic interpretations.

The current study aimed to keep the vignettes as equal as possible regarding the details of the perpetrator. However, in doing so, the history of the victim–perpetrator relationship was not provided. Therefore, future studies could alter the vignettes to include abuse duration, since intrafamilial CSA is often more prolonged due to greater accessibility to the victim (Bagley & Pritchard, 2000), and involves non-physical techniques, such as grooming (Marshall et al., 2015). This added detail would aid in understanding the effect of abusive techniques on participants’ perceptions. This would further raise awareness regarding the disconnect between public perception and reality. Finally, in the present study, the CSA was perpetrated by an adult man. It would prove useful to also investigate how people perceive intrafamilial and extrafamilial CSA perpetrated by a female or an adolescent, and how it compares to the results of this study. Indeed, existing research has demonstrated a variation in judgments toward CSA when perpetrated by a female compared to a male (Bornstein et al., 2007; Rogers & Davies, 2007; Socia et al., 2019b) and when perpetrated by an adult compared to adolescent (Harper & Bartels 2018; Sparks & Wormith, 2021). It would also be interesting to examine whether a “first name bias” had an additional effect on participants’ judgments. Research indicates that, when the name and race of a suspect reflects a black person, participants interpret and remember information about a criminal case in a racially biased manner (Levinson et al., 2007). Since the name used in the vignettes of the current study (Charles) could be seen as a prototypical white name, this may have influenced judgments and so could be an area of future research to examine.

Despite these limitations, the current study has several strengths. For example, although some studies have examined attitudinal responses across differing CSA offenses, the difference tends to be in terms of gender and age (e.g., Harper & Bartels, 2018). This current study focused on known CSA subtypes instead. The few studies that have examined responses to intra- and extrafamilial CSA have done so in a broad sense by referring to how they are categorized. For example, a “stranger” is often used to represent an extrafamilial case (McCoy & Gray, 2007), despite the literature telling us that victims often know their perpetrator, even if they are extrafamilial. Thus, adding the proximity factor enabled this to be accounted for in the present study. Further, the current study compared a biological and sociolegal father, where differing perceptions are less understood (Pullman et al., 2017).

### Implications

The results of this study may offer some useful practical implications, particularly with regards to raising awareness or informing the public (and professionals) about common CSA myths. We found that judgments were more negative and harsher for the extrafamilial cases compared to the intrafamilial ones (despite the offense details remaining identical). Thus, it is possible that these judgments may surface in real-life, for example, during jury decision-making procedures. The current research could, therefore, help prevent or correct such misconceptions. For example, they could be used to create a strategy (e.g., a short educational video) that informs people about the biases that may be at play when making courtroom-based decisions. The findings could also be used to inform public policies around prevention so that they do not reinforce existing misconceptions (e.g., focusing only on “stranger danger”). Similarly, the findings could be used to inform the public about the myths pertaining to CSA so that, if they ever vote for a policy pertaining to sex offence prevention, they may do so in a less bias-driven manner. By extension, clinical and forensic professionals could be informed of the biases held by the public, so that they are aware of them when forming assessment or treatment-based decisions.

## Conclusion

The results of this study suggest that the public’s attitudinal response to CSA differs as a function of the relationship type (intrafamilial vs. extrafamilial) and relationship proximity (close vs. distant). Specifically, men who commit extrafamilial CSA, particularly strangers, are perceived as more dangerous, more pedophilic, and more disgust-inducing than intrafamilial counterparts. This indicates that, when examining public perception of CSA, it is important to account for the heterogeneity of those who commit such offenses. This could have implications for developing educational or awareness raising strategies that can be used to reduce or correct CSA-related biases that may held by, for example, jurors, policymakers, and forensic professionals. Further research should look to expand upon this study by including a distant stepfather condition and other factors, such as different perpetrator genders, specific pre-offense behaviors (e.g., grooming), and offense histories.

## Appendix 1

**Father Vignette:** Charles is a 30 year-old stay-at-home dad. He and his wife have one child. One day, Charles was watching over his child whilst his wife was working in her office. Charles went to the child’s bedroom where they were playing. Charles sat very close to the child and put his arm around their waist as he asked about the game they were playing. He then placed his hand on the child’s knee and started moving his hand up until he was eventually touching the child’s genitals. During this interaction, Charles told the child that he cared deeply for them. Afterward, Charles told the child not to tell anyone about what happened as it was a secret between only them.

**Step-Father Vignette:** Charles is a 30 year-old stay-at-home stepdad. He and his wife have one child. One day, Charles was watching over his child whilst his wife was working in her office. Charles went to the child’s bedroom where they were playing. Charles sat very close to the child and put his arm around their waist as he asked about the game they were playing. He then placed his hand on the child’s knee and started moving his hand up until he was eventually touching the child’s genitals. During this interaction, Charles told the child that he cared deeply for them. Afterward, Charles told the child not to tell anyone about what happened as it was a secret between only them.

**Uncle Vignette:** Charles is a 30 year-old uncle. Charles had only met his nephew a couple of times since he did not live very close. Charles was looking after his brother’s child for the evening whilst the parents worked in their offices. Charles went to the child’s bedroom where they were playing. Charles sat very close to the child and put his arm around their waist as he asked about the game they were playing. He then placed his hand on the child’s knee and started moving his hand up until he was eventually touching the child’s genitals. During this interaction, Charles told the child that he cared deeply for them. Afterward, Charles told the child not to tell anyone about what happened as it was a secret between only them.

**Babysitter:** Charles is a 30 year-old babysitter who has been caring for a married couple’s only child for the past year. One day, Charles was watching over the child whilst the parents were working in their respective offices. Charles went to the child’s bedroom where they were playing. Charles sat very close to the child and put his arm around their waist as he asked about the game they were playing. He then placed his hand on the child’s knee and started moving his hand up until he was eventually touching the child’s genitals. During this interaction, Charles told the child that he cared deeply for them. Afterward, Charles told the child not to tell anyone about what happened as it was a secret between only them.

**Stranger:** Charles is a 30 year-old plumber employed to work in a household with an only child. He has never met the child before but is trusted by the parents to be in the house. The parents were working in their offices in the house and the child was playing in their room. He went upstairs to check the boiler and noticed the child. Charles went to the child’s bedroom where they were playing. Charles sat very close to the child and put his arm around their waist as he asked about the game they were playing. He then placed his hand on the child’s knee and started moving his hand up until he was eventually touching the child’s genitals. During this interaction, Charles told the child that he cared deeply for them. Afterward, Charles told the child not to tell anyone about what happened as it was a secret between only them.
